# Explicit Instruction of Scientific Uncertainty in an Undergraduate Geoscience Field-Based Course

**DOI:** 10.1007/s11191-022-00345-z

**Published:** 2022-05-11

**Authors:** Kathryn M. Bateman, Cristina G. Wilson, Randolph T. Williams, Basil Tikoff, Thomas F. Shipley

**Affiliations:** 1 Department of Psychology, Temple University, 1701 N 13th Street, Philadelphia, PA 19122 USA; 2 Department of Electrical and System Engineering, University of Pennsylvania, 200 S 33rd Street, 201 Moore Building, Philadelphia, PA 19104 USA; 3 Department of Geology, University of Wisconsin-Madison, 1215 W Dayton St, Madison, WI 53706 USA

## Abstract

Understanding and communicating uncertainty is a key skill needed in the practice of science. However, there has been little research on the instruction of uncertainty in undergraduate science education. Our team designed a module within an online geoscience field course which focused on explicit instruction around uncertainty and provided students with an uncertainty rating scale to record and communicate their uncertainty with a common language. Students then explored a complex, real-world geological problem about which expert scientists had previously made competing claims through geologic maps. Provided with data, expert uncertainty ratings, and the previous claims, students made new geologic maps of their own and presented arguments about their claims in written form. We analyzed these reports along with assessments of uncertainty. Most students explicitly requested geologists’ uncertainty judgments in a post-course assessment when asked why scientists might differ in their conclusions and/or utilized the rating scale unprompted in their written arguments. Through the examination of both pre- and post-course assessments of uncertainty and students’ course-based assessments, we argue that explicit instruction around uncertainty can be introduced during undergraduate coursework and could facilitate geoscience novices developing into practicing geoscientists.

## Introduction

Uncertainty pervades all aspects of scientific practice, including data collection, analysis and synthesis, and model building. In the context of science education, Jordan and McDaniel define uncertainty from a cognitive perspective as a “subjective experience of doubting, being unsure, or wondering how the future will unfold, what the present means, or how to interpret the past” which can pertain to individual actors, their environment, or the interactions between them (Jordan & McDaniel, 2014, p. 492). When experiencing uncertainty, scientists are often unsure how to explain something (e.g., phenomenon, data, or anomalous pattern) based on what they currently know (Chen, 2020). Socioculturally, uncertainty is managed through interactions which negotiate the “controversy, conflict, and difference among/between individuals who interpret differently a phenomenon, raw data, or discussed issue” (Chen, 2020, p. 334).

Strategies for learning about, communicating, and managing uncertainty provide scientists with tools to act in the face of uncertainty. This includes support from peers when one voices uncertainty (Jordan & McDaniel, 2014) and through opportunities to exercise epistemic agency during knowledge building practices (Miller et al., 2018). However, the science education community lacks consistent norms to discuss uncertainty. Creating these norms is challenging because it requires acknowledging that one does not know something, articulating that uncertainty, and providing an assessment of the level of uncertainty publicly. Yet, problems that capture the attention of scientists are exactly the kind that are highly uncertain and lack clear, straightforward resolutions. To reason about these wicked problems (Rittel & Webber, 1973), it could be valuable for scientists to share uncertainty around their data and models with each other in the process of making claims. This communication may take the form of statistical metrics of variability in data, and subtleties of language to distinguish the amount of uncertainty in a model (e.g., choosing among theory, hypothesis, and conjecture).

We approach the uncertainty in science from a sociocultural perspective in which science is a cultural process (Latour, 1987) and uncertainty is an inherent part of that process. Uncertainty resides within the people, the products, and the process of doing science and communicating it. The knowledge building process, a complex goal of science, requires us to consider what is known and what is yet to be known. However, in many classrooms, uncertainty is conceptualized as a negative thing that must be minimized (Chen, 2019; Kampourakis, 2018; Lee et al., 2020) rather than a pedagogical tool to enhance knowledge building (Chen & Qiao, 2020).

In this paper, we examine the ways in which explicit instruction in data and model uncertainty supported undergraduate geoscience students in recognizing and explaining uncertainty around the practice of geoscience. We describe the design of an online course, an uncertainty rating scale, and student assessments of uncertainty. Through the examination of both pre- and post-course assessments of uncertainty and students’ course-based assessments, we argue that explicit instruction around uncertainty can be introduced during undergraduate coursework and could facilitate geoscience novices developing into practicing geoscientists.

### Uncertainty in Geoscience

Uncertainty is a feature of all sciences, but uncertainty within the geosciences often stems from geoscience predominantly being an historical rather than experimental science. Although there are some overlaps, historical sciences, including many geosciences, and experimental sciences, like chemistry and physics, employ different methods and data types because the disciplines ask different questions and solve different types of problems (Cleland, 2002, 2013). Geoscience practice is often critiqued by experimental sciences for contending with missing or incomplete data, an inability to create lab-controlled settings, and large temporal and spatial scales (Frodeman, 1995). In some cases, data can be inaccessible so relevant observations cannot be made, or are unrecoverable, lost in the past to natural processes (Bond et al., 2007; Cleland, 2013). For example, most of the rocks on earth are deeply buried and difficult or impossible to access directly. In other cases, unconformities (or temporal breaks in the rock record) indicate parts of the rock record are missing—and no information is available at that location from the missing time interval. Geoscientists can make inferences about what may have occurred (i.e., causes of formations being faults or folds, erosional processes that led to visible structures), but they cannot undo history and confirm what was there.

Furthermore, unlike counterparts in chemistry or physics, geoscientists take a hermeneutic approach, selecting the best tools to do the work on the problem at hand. In many ways akin to educational ethnographers, a field-based geologist must rely on their own prior knowledge, training, and developed skill set to collect and analyze data (Frodeman, 1995). As with the example of unconformity, geologists use the tools (cognitive, social, and physical) they have at hand to decide what data gets collected, how it gets collected, and how it gets interpreted. Each of these phases injects uncertainty into the outputs of the research (Kirch, 2012). Therefore, the social nature of science must also be accounted for in how we communicate science (Oreskes, 2015). Uncertainty in the data that was collected and the model that resulted from the data must be made clear to those who are reading and using the outputs of geoscientific work if the findings are to be useful in decision making and furthering scientific work.

Uncertainty in both the data and model can stem from the geoscientist themselves through their biases. Each geoscientist, when approaching a new phenomenon, brings prior knowledge they have accumulated over time. The content knowledge, experiences, etc. of individual geologists produce differing yet plausible explanations for the same phenomenon. Bond et al. (2007) demonstrated this effect in interpretation of geophysical data, such that only 25% of experts correctly identified the three major geologic features present in a data image. Expert interpretations tended to be based on their dominant area of expertise, interpreting the data in a context (e.g., a tectonic setting such as extensional regimes) with which they were most familiar. In short, when making decisions under conditions of high uncertainty, people tend to rely on prior knowledge to intuitively guide choice (Bond et al., 2007). Although this approach can offer satisfactory and efficient solutions for many decisions, it can sometimes yield less-than-optimal choices, collectively referred to as human decision biases. Previous research has demonstrated the influence of such biases in geologic interpretation and decision making (Alcalde et al., 2017a, 2017b; Wilson et al., 2019; Bond et al., 2007; Polson & Curtis, 2010; Rowbotham et al., 2010; Taylor et al., 1997). A common language for uncertainty documentation and communication may support scientists, and geologists specifically, in overcoming these biases in decision making (Wilson et al., 2019; Macrae et al., 2016; Milkman et al., 2009; Soll et al., 2014).

A common language to characterize scientific uncertainty provides a mechanism for an individual to communicate how certain or uncertain they are to another. Awareness of uncertainty has been shown to improve the quality of scientific arguments (Lee et al., 2019). Thus, the development of an explicit typology or taxonomy of uncertainty may well improve geologic practice (Fischhoff & Davis, 2014). Although an uncertainty typology developed for geosciences field work may be adaptable to other sciences, one uniform uncertainty categorization scheme is unlikely to serve all sciences because data types and their relevant uncertainties differ so widely across disciplines. The development of appropriate discipline-specific typologies, however, could facilitate communication by bridging epistemological and cultural differences between disciplines (Doyle et al., 2019). Existing uncertainty typologies of geoscience focus on capturing analytical uncertainties associated with instruments or the range of uncertainties associated with modelling of natural processes (Caers, 2011), and only indirectly on the human role in interpreting observational data.

Geologic field-based data, when translated to models, such as geologic maps, are often without any indication of the uncertainty of that model (Pérez-Díaz et al., 2020). Some maps have some existing conventions, but the conventions lack specificity of how certain the geologist was with respect to any feature. Solid, dashed, and dotted lines between units—for both faults and depositional contacts—convey levels of decreasing certainty in specific features (for example, see Ernst & Hall, 1987 and Bilodeau & Nelson, 1993). However, the maps do not include information about the uncertainty of the observations from which the model was created. Park et al. (2013) developed a way to communicate uncertainty for reservoir development using massive data collection and computer model analysis, but these tools are not generally available when conducting field-based research. A nuanced way of communicating the critical aspects of uncertainty is needed.

### Role of Science Education in Uncertainty

Science education provides opportunities for learning about uncertainty in scientific practice and communication. Calls for explicit instruction around the role of uncertainty in science have been made for over 20 years (Chen & Klahr, 1999). However, science theory and data are often conveyed as “known,” with the implicit assumption that data and models exist without uncertainty. In classrooms, “doing school” privileges the certain over the messiness inherent with authentic science practice (Jiménez-Aleixandre et al., 2000; Manz, 2015). What is being communicated to students in these authoritative science classrooms, implicitly or explicitly, is that the doing of science involves collecting *certain* data which leads to *certain* theories. However, many aspects of science are inherently uncertain; science is a process with an imperfect, evolving sense of reality (Metz, 2004). Fortunately, approaching scientific uncertainty as a cultural facet of science makes it one that can be learned and taught in classrooms (Crawford et al., 2000).

The Framework for K-12 Science Education (National Research Council, 2012) and the Next Generation Science Standards (NGSS Lead States, 2013) both call for teaching uncertainty, but that has not led to it being included in teaching practices. The NGSS presents scientific uncertainty as something that can be minimized, related to error, and complexity (Buck et al., 2014), rather than an inherent part of science and a stimulus for creativity (Pollack, 2003).

If teaching uncertainty is important, a clear model that articulates uncertainty is needed to teach it well (Kirch, 2012). Although some have posed potential models for scientific uncertainty (i.e., Costanza & Cornwell, 1992; Fischhoff & Davis, 2014), there is currently no universally accepted, domain-general model. Models have been generated to support undergraduates in specific fields such as climate change (Brewer & Gross, 2003), but these are focused on the statistical analysis and the inherent noise in the data. Further, of the models posed, they do not afford students with a way to discuss the level of uncertainty experienced by other scientists in a uniform way.

Explicit instruction around uncertainty bolsters sensemaking and argumentation around the phenomenon under investigation (Buck et al., 2014). Manz (2015) posits that argumentation should take a central role in science education, with uncertainty being central in those arguments as learners work towards stabilizing scientific knowledge. This approach is fostered through situating the learning in a real-world, complex phenomenon (Manz & Suárez, 2018). For example, Pallant & Lee (2015) situated student learning in publicly released data from NASA’s Earth Observatory, including Vostok ice core data and climate models, to support students in crafting scientific arguments. As an explicit part of the curriculum, students evaluated the uncertainty in their argument. However, instruction to students on uncertainty or how to write about it in their arguments was not part of the instructional design. Here we offer an approach to uncertainty instruction and use grounded in science practice, inclusive of uncertainty in data and models that also offers a language to compare and communicate uncertainty.

**Fig. 1:**
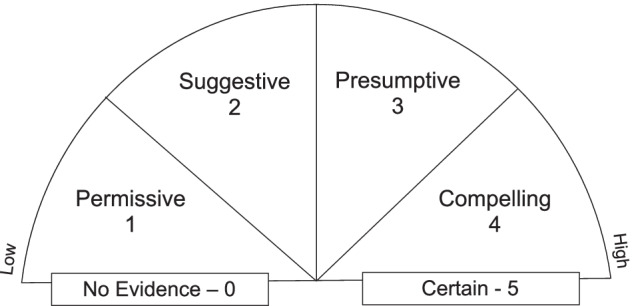
Uncertainty rating scale

In designing a course for undergraduate geoscience majors, our team hypothesized that a tool to explicitly quantify uncertainty of observational data could support geologists in the field in making decisions and claims about the geology of the area. We developed a six-category uncertainty scale (Fig. 1), that can be incorporated into a geologists’ practice to provide a way to characterize uncertainty in field-based data which was then explicitly taught to undergraduate geoscience majors during the final week of a virtual capstone course in the summer of 2020. In our study of the course’s use of the uncertainty rating scale we asked, “In what ways does quantifying data and model uncertainty in an undergraduate geoscience course influence the ways geoscience majors explicate the roles of uncertainty in the practice of geoscience?” We further break this question down into two subquestions:

- In what ways does explicit instruction around data and model uncertainty inform undergraduate geoscience students’ attributions of why there might be differences among experts in their explanations of phenomena?
- In what ways does explicit instruction around data and model uncertainty inform undergraduate geoscience students’ construction of an explanation of a geoscience phenomenon?

## Methodology

### Context

Data for this study were collected as part of a virtual field camp course during the year 2020, when in-person field camps were generally cancelled because of the pandemic. The field camp was taught as a series of online modules over six weeks. We developed materials for the final week, which focused on the Sage Hen Flat pluton and surrounding rocks in the White Mountains of eastern California (Table 1). Although the module was focused on how the pluton was intruded into the surrounding Precambrian and Paleozoic strata, uncertainty was also a focus of the final week. The work was arranged so that the students built up to evaluation of how the pluton was emplaced and what was the tectonic history of the area. Data uncertainty was presented on day 2 and model uncertainty on day 3. At the culmination of the week-long module and course, students constructed a scientific argument to display their understanding of what was happening in the field area. In these following sections, we describe in more detail the uncertainty rating scale used in the course, the way in which uncertainty was taught to the students, and the assessments used to measure student learning of both geology and uncertainty.

**Table 1:** Syllabus outline for Sage Hen pluton/uncertainty module

Day	Instructional activities	Format
1	Pre-assessment	Individual
	Overview of module	Whole group/lecture
	Reading on uncertainty in the practice of science (Cargo Cult Science)	Individual
	Video overview of White Mountains	Whole group/lecture
	Google Earth exploration of White Mountains	Individual
	Determining rock type and stratigraphy in Google Earth	Individual
	Develop stratigraphic section	Individual
2	Introduction of granitic fabrics	Whole group/lecture
	Introduction to data uncertainty	Whole group/lecture
	Student-led exploration of granitic AMS fabrics. Focus on role of dispersion	Individual
	Student-led exploration of mineralogy. Focus on role of interpolation	Individual
	Student-led exploration of microstructures. Focus on role of interpretation and inference	Individual
3	Model uncertainty	Whole group/lecture
	Cross-section self-evaluation	Individual
	“Using uncertainty in workflows”—interpretational uncertainty	Small group
	Evaluation of maps using model uncertainty	Individual
	Creation of geologic map	Individual
4	Overview of gravity data	Whole group/lecture
	Instructor-led discussion of uncertainty in gravity data	Whole group/lecture
	Sketch cross-section of Safe Hen Flat pluton	Individual
	Structure contour mapping of lower contact of Sage Hen Flat pluton	Small group
	Cross-section through Sage Hen Flat with correct pluton geometry	Individual
5	Introduction to faults in Sage Hen Flat pluton	Whole group/lecture
	Comparing faults in basalt and granite in Sage Hen Flat pluton with uncertainty ratings assigned by students	Small group
	Situating the faults in context (time, type, locations)	Individual
	Creation of final cross-section	Individual

#### Uncertainty Rating Scale

To normalize the students’ discourse around data and model uncertainty, an uncertainty rating scale was designed loosely based on Dott’s “evidence meter,” a classroom tool used by Dr. Robert Dott (University of Wisconsin—Madison), who had adapted it from one used by Dr. Preston Cloud (University of California—Santa Barbara). The uncertainty rating scale (Fig. 1) has two endpoints—“No Evidence” and “Certain”—that bound the intermediary levels where much of scientists’ data and model uncertainty lies. There are four intermediate levels of uncertainty—*permissive*, *suggestive*, *presumptive*, and *compelling*—which are terms drawn from geology practice. Permissive, the least certain, suggests a particular property or inference cannot be ruled out, but it is not the only available solution. Suggestive indicates that the scientist has positive evidence for a particular property or inference, but that the evidence also allowed for the possibility of other properties or inferences. Presumptive assumes the proposed property or inference is more likely right than wrong in the absence of any additional evidence. Finally, compelling strongly supports a property or inference based on high levels of evidence available. The choice of four intermediary levels provides a familiar quartile scale that can be associated with relative likelihoods (0–25, 25–50, 50–75, 75–100) that will be familiar to scientists and the general public. A similar rating scale was used for model uncertainty, with one difference: There is no “certain” rating for models, because they are always subject to revision as new data or understandings become available.

#### Course Design

The virtual field course took place over 6 weeks, with each week capturing different aspects of geoscience field work. The final week focused on the Sage Hen Flat pluton in the White-Inyo Mountains of California and the uncertainty rating scale. The full module, inclusive of all data sets and guided learning, is hosted online by the Science Education Resource Center (Tikoff et al., 2020), and is part of the National Association of Geoscience Teachers Online Field Experiences Exemplary collection. Student participants were 34 undergraduate students from six public universities across the USA who enrolled in a 6-week capstone course designed to prepare them for careers in the geosciences. The capstone is traditionally field-based, but due to the COVID-19 pandemic all activities occurred online in 2020.

The Sage Hen Flat pluton provided an excellent context to introduce the uncertainty rating scale as two published maps of the area (Bilodeau & Nelson, 1993; Ernst & Hall, 1987) present different interpretations of the same geology. The format of the course follows teaching strategies suggested by Manz & Suárez (2018) that allowed uncertainty to be incorporated in the classroom as the module was embedded in a complex phenomenon, created opportunities for iteration on ideas, and leveraged variability in methods. Students were introduced to the two maps of a complex geologic area at the start of the week and then iteratively created models (maps and cross-sections) of the area using various data sets from the same location over the week. At the end of the week, students created a final written argument and accompanying geological map in favor of a new hypothesis or one of the existing models. The syllabus outline for the course is provided in Table 1. Over 5 days of instruction, students oscillated between synchronous interaction with instructors (whole group/lecture) and peers (small group), and asynchronous independent work (individual).

**Fig. 2:**
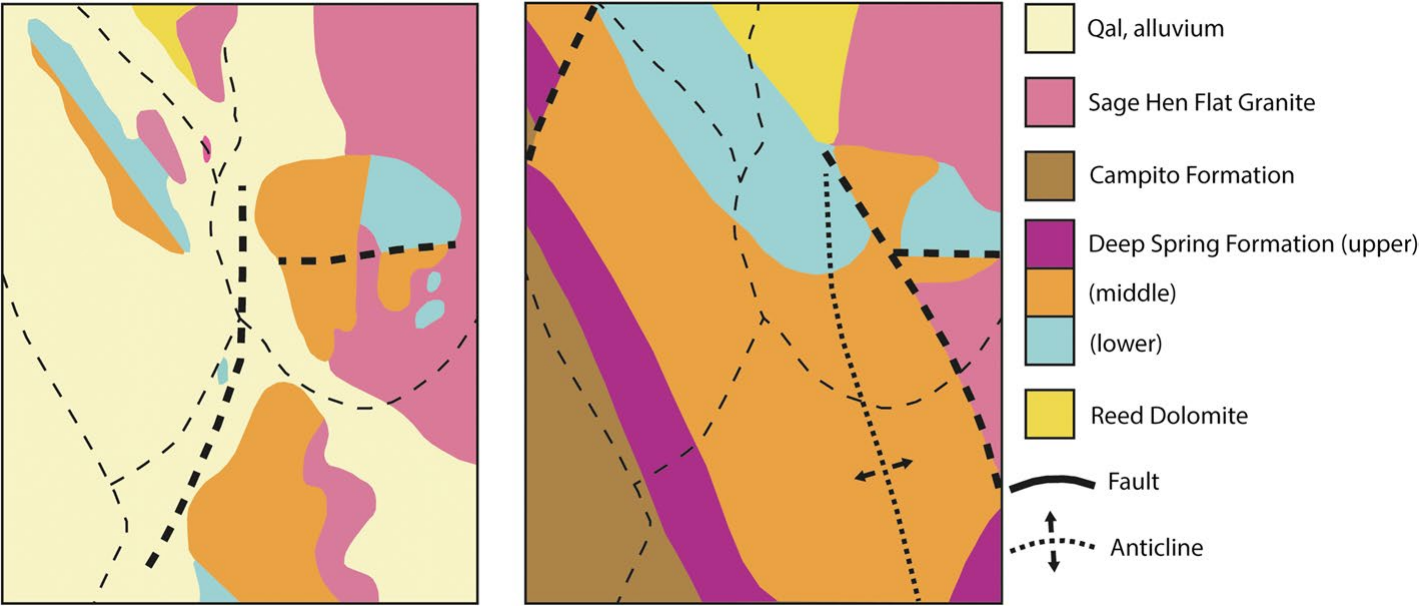
Simplified drawings of the Ernst & Hall (1987) and Bilodeau & Nelson (1993) maps of the Sage Hen Flat pluton shared with students inthe Sage Hen Flat module. Maps modified from a student project by L.D. Wilson, J.D. Higdon, and J.A. Davidson; Courtesy of A. Glazner; Color versions available online, see Tikoff et al., (2020)

We chose to situate the module in the Sage Hen Flat pluton for both geological and pedagogical purposes. Geologically, the Sage Hen pluton served our purposes well because there are existing maps, created by professional geologists in the latter half of the twentieth century (Bilodeau & Nelson, 1993; Ernst & Hall, 1987), with conflicting interpretations (Fig. 2). Pedagogically, these discrepant maps allowed us to situate the learning in a complex phenomenon with a large and diverse data set which would raise uncertainty for students and provide opportunities for them to sense-make and reason (Furtak & Penuel, 2019; Manz & Suárez, 2018). The maps differ primarily in their interpretation of the relationship between the pluton and the surrounding rocks: The map created by Ernst & Hall (1987) depicts a fault between the pluton and adjacent rock. The map created by Bilodeau & Nelson (1993) interprets the contact between the pluton and adjacent rock as being the result of an intrusion. Other areas of the two maps also differed, reflecting differences in the inferred history of the pluton. The geological maps indicate some level of uncertainty about the location of contacts between rock units (the boundary between two volumes of rocks with differing origin), which presumably informed the geologists’ interpretations. What is unknown from the maps is why and at what level the geologists were uncertain in the location of these contacts. Critically, the maps were not presented in the module in a way that implied one was “correct” and the other “wrong.” The two conflicting maps allowed students to see that professional geologists could have different, yet plausible, interpretations of the same data / observations. Thus, the exercise offered students an opportunity to consider the map-makers’ potential uncertainty, and emphasized that maps were models and interpretations not evidence.

#### Sage Hen Flat Uncertainty Module Outline

The first day of the course was devoted to familiarizing students with the context of the White Mountains and introducing uncertainty through Feynman’s speech on Cargo Cult Science (1974). In Cargo Cult Science, Feynman describes a tribe of South Pacific Islanders who have designed runways for planes to land in hopes that World War II era cargo planes will bring them supplies as they had in the 1940s, but alas, planes do not come, even though the people on the island have replicated the processes from before. His point to the audience was that if there is something amiss in your experiment, your science, you need to try to explain it. The same can be said regarding uncertainty—we need to articulate the places we just do not know and cannot be sure.

**Fig. 3:**
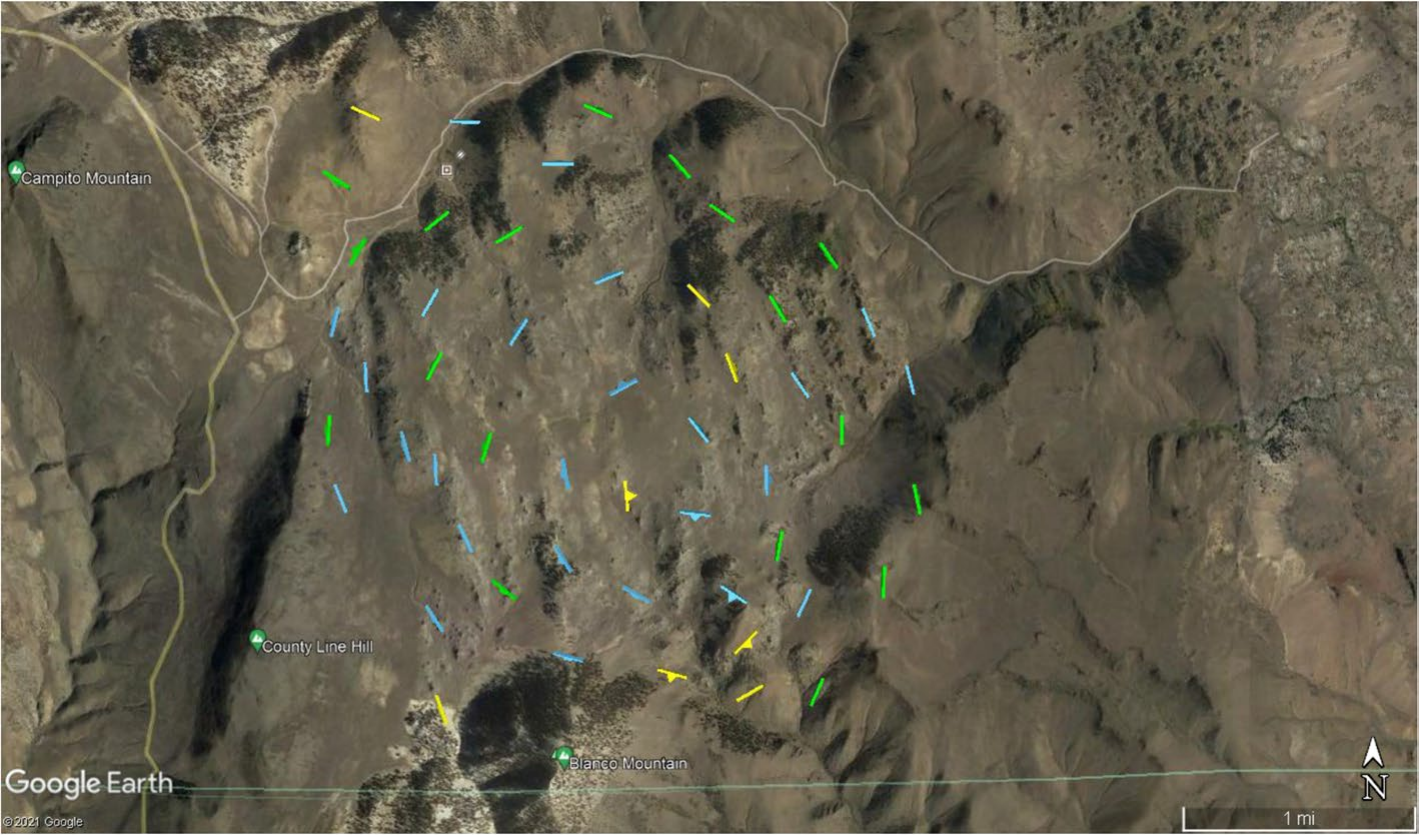
Screen shot of Google Earth image of magnetic foliation data on day 2 of the module which includes color-coded uncertainty ratings where compelling = blue symbols (less than 10° of dispersion of all of the samples; presumptive = green symbols (less than 20°of dispersion of all of the samples); and suggestive = yellow symbols. (less than 45°of dispersion of all of the samples)

On day 2, students engaged with the science of granitic fabrics and the concept of data uncertainty (see 2.1.4), which were then applied to individual activities with data from the White Mountains around dispersion, interpolation, and inference. Students were posed questions as they explored data layered in Google Earth that included uncertainty ratings (Fig. 3) such as, “For the evaluation above, are you evaluating your data? Or, are you evaluating a model of your data? Explain your answer.” In another question during their examination of interpolation of minerology data, students were asked, “How confident are you drawing the boundaries between the two zones? Go ahead and draw them. Explain your uncertainty in drawing those contacts.” This required students to make connections between their claims and the amount of uncertainty they had in those claims, inclusive of the data uncertainty provided in Google Earth. Finally, students created a cross-section, a model of the rock structures in the Sage Hen Flat pluton based on the data currently available to them.

**Fig. 4:**
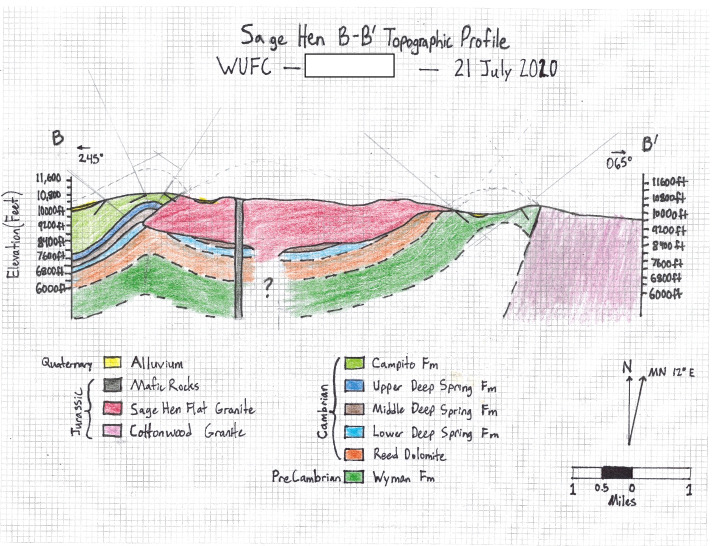
Exemplar student cross-section from day 4 of the module. Student demonstrated awareness of uncertainty by including a question mark in an empty area and dashed lines in the sketch

On day 3, students were given direct instruction on model uncertainty (see 2.1.4) and then students evaluated classmates’ cross-sections from the previous day, explored new data in Google Earth, and constructed new geologic maps incorporating the new day’s data with previously available data. Then they made a first approximation of a geologic map of the area based on the evidence they had so far. On day 4, an isopach map (the thickness of the unit below the surface)—derived from gravity data—was provided and an explicit discussion of the uncertainty in the data set was held. After these discussions, students created cross-sections along a north–south transect and an east–west transect. Next, they worked in their small groups to use the available evidence to create a structure contour map, and then independently worked to create a second cross-section intended to revisit their earlier ideas in light of new evidence (Fig. 4).

On the final day of the module, evidence of faults in the Sage Hen Flat was presented, adding to the complexity of the geology in the area. In their small groups students compared the fault information in the different rocks and assigned the uncertainty ratings using the uncertainty rating scales presented earlier in the week by instructors. Finally, students created two final products to demonstrate their understanding of the geology of the White Mountains and Sage Hen Flat pluton, as well as uncertainty—a written narrative of the geologic history of the Sage Hen Flat pluton and a cross-section map that mirrored the transect on the Bilodeau & Nelson (1993) map.

#### Structure of Explicit Uncertainty Instruction

**Fig. 5:**
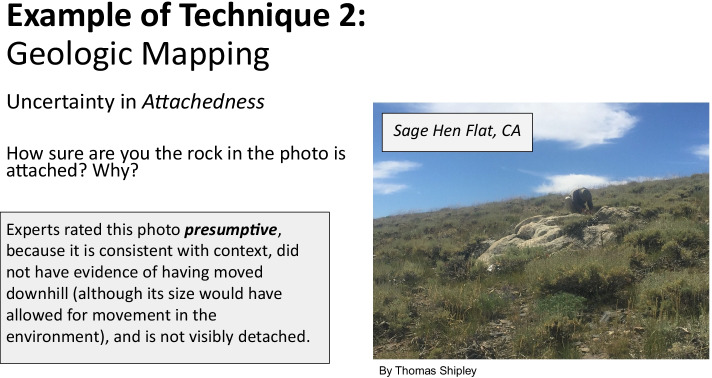
Screenshot of slide from the uncertainty module instruction on “attachedness”

We designed the Sage Hen Flat module to teach students to use the uncertainty rating scale through worked examples, drawing on cognitive apprenticeship frames (Brown et al., 1989). Within the Sage Hen Flat module, we situated the uncertainty rating scale in a complex, real-world problem to solve, with complex data to align with the experience professional geologists have in the field. To give students opportunities to understand and practice the rating scale, we first provided geologic information about which they needed to sense-make. After students had an opportunity to think through the problem on their own, they were provided with the thinking of an expert geologist to model the use of the uncertainty rating. For example, students were asked to rank uncertainty regarding “attachedness,” or, how sure are they that a given rock is attached to a larger rock mass in the subsurface given a photograph from the field (Fig. 5). Students used the uncertainty rating scale to try their hand at making their own assertion. Next, we provided an expert’s uncertainty ranking as well as an explanation of why they gave that explanation. For example, using a photograph of a rock visible among the grass in the Sage Hen Flat, students read, “Experts rated this photo *presumptive*, because it is consistent with context, did not have evidence of having moved downhill (although its size would have allowed for movement in the environment), and is not visibly detached” (Fig. 5). In these examples, we provided students with access to the internal thought process that led geologists to their recorded level of uncertainty. Making the geologist’s thinking visible helps the students to gain more understanding of the internal workflow of the geologist and to develop this workflow for themselves (Brown et al., 1989).

To develop students’ understanding of the interplay of model and data uncertainty, the module included videos of geologists in the field to show explicitly how expert geologists reasoned about this interplay. In these videos, geologists in the field worked through different potential interpretations of the geology in the area, making transparent the (often hidden) negotiation of meaning that occurs in the head of geologists when grappling with uncertainty in the data and the developing model.

#### Assessments of Uncertainty

Students’ understanding and application of uncertainty were evaluated with an assessment of uncertainty (before and after the module) and a final written argument, both designed by the authors. The final argument was graded by the instructor and included in the students’ course evaluation. The before and after assessments were part of the course activities but not graded.

**Fig. 6:**
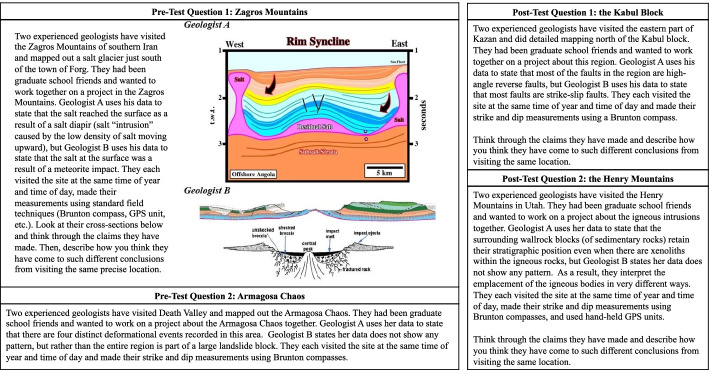
Questions from the assessment of uncertainty

To capture how conceptions of scientific uncertainty changed from experience with our module, we asked participants to complete an assessment of uncertainty (Fig. 6) before and after the module. In the assessment, participants were provided with two scientific scenarios, wherein two expert geologists with comparable levels of training reach different scientific conclusions from visiting the same site. In the first scenario, the experts have different interpretations of data but both offer different interpretations of the geologic process(es) that produced them, i.e., there is a signal in the noise. In the other scenario, one expert detects an interpretable pattern (signal) and one expert decides that there is no pattern (just noise). The purpose of the scenarios was to assess participants’ understanding of the varying sources of uncertainty that could lead to trusted experts coming up with fundamentally different interpretations of the geologic process that formed the geology observed today. The scenarios were general enough that they did not require prior knowledge of the geological area in the scenario to respond.

For each scenario, participants described in an open response what factors they might consider when looking at the claims and data in the scenario and used a 5-point Likert scale (1—very unlikely, 5—very likely) to express the likelihood of different factors that could have contributed to conclusions reached by geologists (Fig. 6). Questions 2 and 3 related to operational uncertainty (data quality, measurement error) and question 4 related to epistemic uncertainty (natural variability). Questions 5–7 related to uncertainty stemming from the socially constructed nature of the scientist’s conclusions (prior assumptions in data collection and data interpretation, and sub-discipline prioritization).

In addition to the assessment of uncertainty, instructors assessed students’ understanding of the Sage Hen Flat module through a final geological map and geologic history. The map provided students’ understanding of the geology of the area considering the data provided throughout the module. The report was a two-to-three-page written argument in which students compared the two previously constructed maps (Ernst & Hall, 1987 and Bilodeau & Nelson, 1993) of the area and critiqued them considering the new data provided during the module. Using the data set collected from the module, students critiqued the maps in relation to the map they had generated. Students were not explicitly asked to provide uncertainty rankings in these reports. Thus, the prevalence of the concept within student’s justifications provided an opportunity for the team to examine the ways in which the uncertainty rating scale had become part of the students’ scientific practice.

### Analysis Methods

We use qualitative and quantitative data analysis to assess both the development of the student’s thinking about uncertainty and the ways students used uncertainty in their geologic practice. First, students’ Likert scale ratings on the assessment of uncertainty were analyzed to evaluate changes in their thinking during the course. Next, using grounded theory approaches (Strauss & Corbin, 1997), students’ short answer responses on the assessment of uncertainty were analyzed to develop themes in the ways students discussed uncertainty in their analysis of the scenarios. Students’ final report arguments were coded to determine if students incorporated uncertainty into their narratives and if so, how. In this section, we will elaborate on the method of these analyses.

#### Assessment of Uncertainty—Quantitative Analysis

In the assessment of uncertainty taken by students before and after the Sage Hen Flat model, students’ Likert scale ratings were grouped for analysis as data-based factors (measurement error, data quality, natural variability) and model-based factors (sub-discipline prioritization, prior assumptions in collecting and interpreting data). Average ratings were computed for data-based and model-based factors for the pre-test and post-test, and then the difference score of the post–pre averages was taken for each participant in each scenario. We anticipated that students would initially report greater likelihood of data-based factors, since to the extent students have been exposed to scientific uncertainty, it is likely to be data uncertainty. After exposure to the module, we expected students to report reduced likelihood of data-based factors, and increased likelihood of model-based factors.

One-tailed, one-sample *t*-tests were run to determine if difference scores were significantly different from zero, which would indicate a significant change in ratings occurred between the pre and post assessments. For data-based factor difference scores, we tested to see if scores were significantly less than zero, which would indicate participants reported a reduced role of data uncertainty from pre to post-test. For model-based factor difference scores, we tested whether scores were significantly greater than zero, which would indicate participants reported a greater role of model uncertainty from pre to post-test.

#### Assessment of Uncertainty—Qualitative Analysis

Our approach to qualitative analysis applied grounded theory approaches (Strauss & Corbin, 1997) to descriptively code (Saldaña, 2021) assessment uncertainty short answer responses to look for ways students discussed uncertainty in their responses. This process was descriptive, not interpretive, summarizing the answers of each student, adding to the set of codes until saturation. This set of 28 codes was processed through the constant comparative method (Strauss & Corbin, 1997) to create abstractions of similar claims. This resulted in a set of five codes generated from student responses as to what factors students felt they would consider when looking at the claims and data in the posed scenarios (Fig. 6; Table 2).

**Table 2:** Codebook for assessment of uncertainty responses for questions in Fig. 6

Code	Definition	Example Student Response to “What factors might you consider when looking at both claims and data?”
Attributed to features of the geologist	Differences in outcomes of scenario derive from differences inherent to the geologist (i.e., background, expertise, training)	“Their individual fields of expertise, their previous work, if their data is the same or if there's a difference there, basically anything that could inform their opinions.”
Requires more geoscience information	Determining reasons for differences in scenario outcomes are unable to be determined without further geoscience information, such as data not mentioned in the scenario	“GPS information along with variations in the strike and dip data.” “Also I would be interested to see what units were placed well and if there was faulting in the area.”
Wanting to see all/raw data	Determining reasons for differences in scenario outcomes are unable to be determined without seeing the collected data before interpretation by the geologists	“Their field notes, locations of measurements, sketches.” “I would like to see what data both geologists collected and compare that first before looking at their interpretation.”
Aspects of instrumentation	Differences in scenario outcomes are attributed to errors in the data collection instrumentation (i.e., uncalibrated compasses)	“Brunton settings of each geologist”
Uncertainty	Responses specifically mention uncertainty in attribution of differences in outcomes of scenario	“What field data do they have, and how certain are they about the various data. How certain are they of their models?”

Students’ responses were coded as follows: (1) attribution to features of the geologist (training, sub-disciplines, biases); (2) requiring more geoscience information (context, history of the area); (3) wanting to see the data (what was collected, where, how); (4) aspects of the instrumentation (calibrations, condition of instrument); and (5) uncertainty (Table 2). Although in some instances students linked the uncertainty to a feature of the geologist, this was coded separately from the more ostensibly fixed attributes of the geologist, such as training. In these instances, uncertainty was more in line with a geologist’s way of thinking than something the geologist was.

#### Final Report—Qualitative Analysis

Final reports were coded to determine if students’ reports included uncertainty and the uncertainty rating scale in the narratives. These final reports are treated as artifacts, reflective of the authors and their perspectives (Hammersley & Atkinson, 2019), in this case, their perspectives on uncertainty in the practice of science. To first analyze these reports, all reports were read by the first author who wrote short analytic memos after each student’s report summarizing an initial interpretation of the student’s use of uncertainty. From these analytic memos, holistic codes were developed (Saldaña, 2021). These codes were informed by our understanding of uncertainty, an inherent part of the people, products, and processes of science, but were open to the language students used and ways in which they used uncertainty in non-normative ways.

**Fig. 7:**
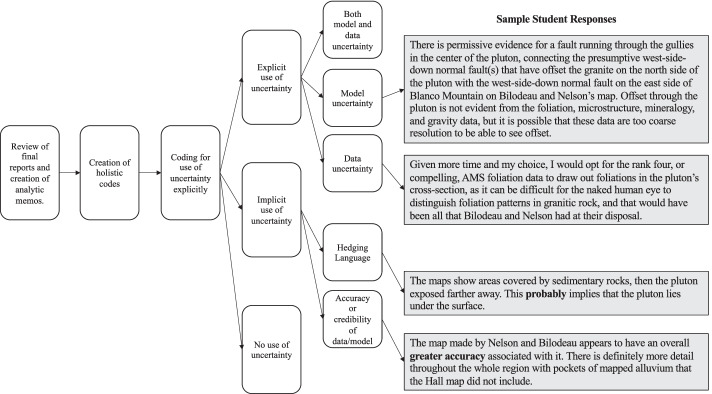
Analytic map of analysis of students’ final reports

With a large set of codes in hand, the initial code set was applied to the full text of the final reports. First, final reports were coded as mentioning uncertainty explicitly or not. Next, students’ final reports that mentioned uncertainty were coded for the type of uncertainty discussed: model uncertainty, data uncertainty, or both. In a final coding cycle, those that did not mention uncertainty were examined for references to uncertainty that were less explicitly such as hedging language and discussion of accuracy and credibility of either data or models. Analytic memos were written at the conclusion of each cycle of analysis. A map of this process was created, inclusive of student quotes (see Fig. 7).

#### Themes in the Qualitative Data

The coding of both the assessment of uncertainty and the final report were examined in tandem to compare students’ use of uncertainty in the final report with the ratings on the uncertainty assessment. This round of coding relied on the first author’s coding memos (Maxwell, 2013) from coding of the assessment of uncertainty and the final reports. Emergent themes were generated through the iterative reading of the coding memos following grounded theory approaches (Strauss & Corbin, 1997) for constant comparison of emerging themes in the data.

#### Validity of Qualitative Data

Following in the qualitative tradition, reliability of the qualitative data was not assessed, opting instead for measures of validity (Maxwell, 2013). Multiple measures were put into the design and communication of the study to bolster the validity. First, multiple sources of data regarding uncertainty were analyzed to produce the findings (Maxwell, 2013). This included quantitative measurements and qualitative responses to pre/post-course assessments of uncertainty and final reports designed to be part of the course. Second, the first author created analytic memos throughout the coding process which were reflected upon and used to iterate on the codes and expose potential biases emerging during the analysis process. Third, systematic coding of the data (Miles & Huberman, 1994; Strauss & Corbin, 1997) was conducted for both the assessment of uncertainty and final reports, outlined in Sects. 2.2.2 and 2.2.3, including Tables 2 and 3 and Fig. 7. Fourth, quasi-statistics (Becker, 1970; Maxwell, 2013) are shared in the findings (Table 3; Sect. 3.2) to support the variation of codes within the sample. Finally, active discourse occurred among authors during the analysis and writing process. The different perspectives (geology, cognitive science, and education) created a triangulation of theoretical approaches (Maxwell, 2013) to enhance the validity and trustworthiness of the final product.

**Table 3:** Students’ (*N* = 34) coded responses during the assessment of uncertainty. Students’ responses included multiple codable answers, so columns do not add to 34. Questions can be found in Fig. 6

	Pre Q1—different interpretations	Pre Q2—pattern/no pattern	Post Q1—different interpretations	Post Q2—pattern/no pattern	Sum
Uncertainty	0	0	7	6	13
Attributed to geologist	9	6	10	15	40
Requires more geoscience information	21	16	8	6	51
Would need to see raw data	8	9	11	13	41
Instrumentation errors	0	2	2	0	4

## Data and Analysis

Analysis of the assessment of uncertainty and the final report revealed that students’ views of science practice had shifted from one in which errors were the sources of uncertainty to being more accepting of inherent uncertainty in data and models. In this section, we describe the data analysis from the three assessments (pre-course assessment of uncertainty, post-course assessment of uncertainty, and final report) and their support of this claim.

### Assessment of Uncertainty

#### Quantitative

**Fig. 8:**
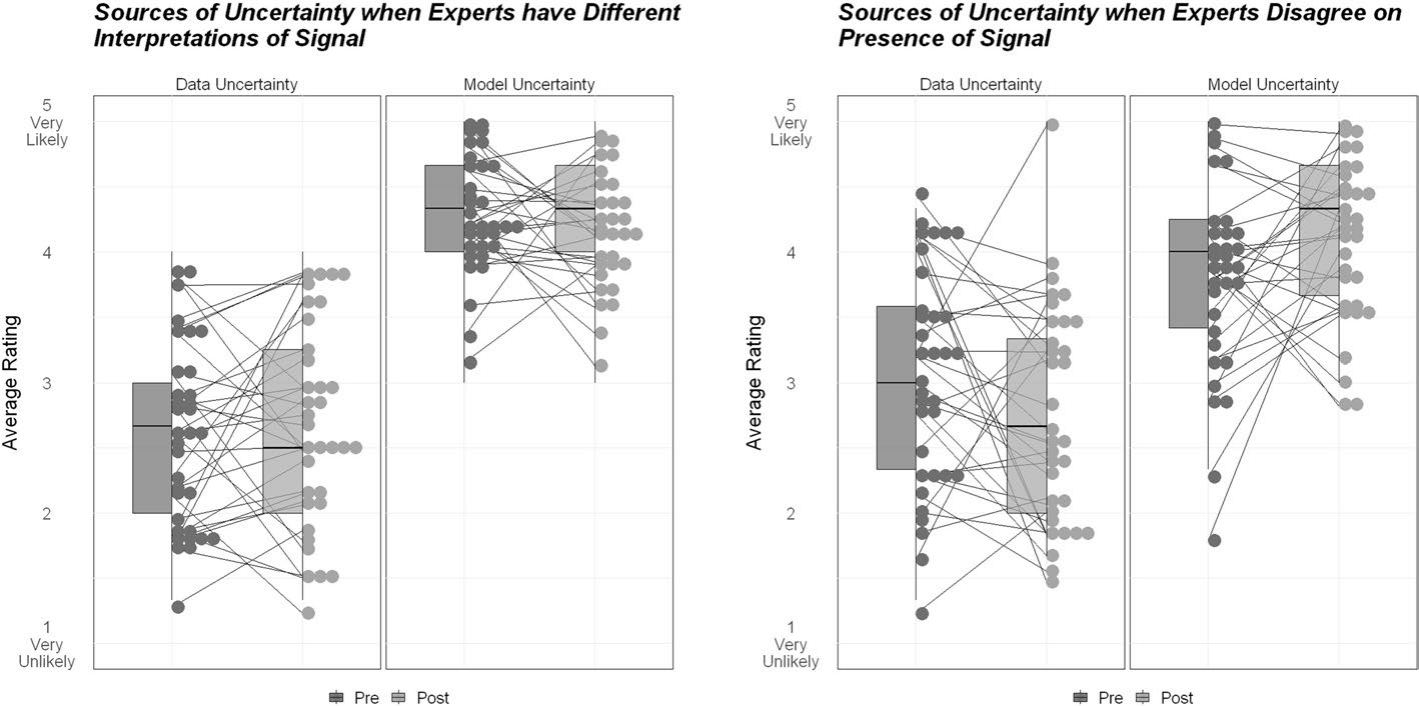
Pre-post-assessment changes in average likelihood ratings for data-based versus model-based sources of uncertainty for different scenarios: (**a)** when experts agree on presence of signal but have different interpretations, (**b)** when experts disagree on presence of signal. Box plots and dots show distribution of ratings, with lines connecting participants’ responses from pre-test to post-test

Across both scenarios, students report an overall lower likelihood of data-based sources of uncertainty contributing to different scientific conclusions, relative to model-based sources (see Fig. 8). This was contrary to our expectations that students would have greater exposure to data uncertainty and suggests that students came to the module with an existing understanding of how differences in mental models can lead experts to reach fundamentally different conclusions.

In the first scenario where experts have different interpretations of data but both think something happened, i.e., there is signal in the noise, there was no statistically significant change in data-based uncertainty ratings, *t* (33) = 0.69, *p* = 0.75, from pre-assessment (*M* = 2.54, *SD* = 0.7) to post-assessment (*M* = 2.64, *SD* = 0.75), or model-based uncertainty ratings, *t* (33) =  − 0.26, *p* = 0.60, from pre-assessment (*M* = 4.28, *SD* = 0.49) to post-assessment (*M* = 4.25, *SD* = 0.51). However, in the second scenario where experts disagree on the presence of a signal, participants’ average ratings of the likelihood of data-based sources of uncertainty are significantly reduced from pre (*M* = 3.04, *SD* = 0.79) to post (*M* = 2.76, *SD* = 0.85), *t* (32) =  − 1.64, *p* = 0.05, while ratings of the likelihood of model-based sources of uncertainty significantly increased from pre (*M* = 3.84, *SD* = 0.76) to post (*M* = 4.13, *SD* = 0.61), *t* (32) = 1.95, *p* = 0.02. This suggests that the module did have some impact on students’ thinking about data-based versus model-based uncertainty, specifically in situations where one expert detects an interpretable pattern (signal) and one detects no pattern (noise).

#### Qualitative/Constructed Responses

In both the pre- and post-tests, students were asked “What factors might you consider when looking at both claims and data?” (Fig. 6). Students only reported uncertainty in responses after interacting with the modules (Table 3). In the pre-test, students more often reported they would want more geological information, such as context, history, or extra data not already collected. This was far less frequent in the post-test data, where in addition to requesting uncertainty information, students more frequently wanted to see the data collected by geologists and/or the processes by which they were collected. There was a slight increase in students stating they would need to know more about the geologists such as their backgrounds and their biases entering the data collection process. In summary, students were less interested in trying to fix uncertainty by collecting more data, and more interested in understanding where the uncertainty originated to help make decisions about trusting scientists’ claims.

### Final Reports

We analyzed the final reports associated with students’ map models of the Sage Hen Flat pluton, the summative assessment of the module, to see if students referenced the uncertainty rating scale, and if so, how. Of the 32 reports completed in sufficient detail to be analyzed, sixteen explicitly used the uncertainty rating scale while another six students made references to uncertainty impacting either their claims or the data. Of those who explicitly reference the rating scale, only two used the rating scales to discuss both model and data uncertainty. These students reported using the uncertainty ratings they either assigned or had been provided for the data to make decisions about what data to include in their models. Students also drew upon the multiple data sets from the Sage Hen Flat module to assign uncertainty ratings to their final maps, an example is shown here.

“When placing faults, I looked for areas of unit offset, valleys, and the presence of faults on a published map. I assessed my level of certainty for each fault proposed from the field data. After assigning each fault a permissive, suggestive, or presumptive certainty level, I added only the presumptive faults to my model. I then compared my chosen faults to the faults systems to the north and west in order to determine the fault type. [...] The unit contacts in my model are generally presumptive and come from compelling field data with the comparison of multiple presumptive mapped contacts.”

This student describes in detail how they were able to evaluate the certainty of the different faults in the existing maps and use that information to compare across the different models they were provided. They also used “compelling” field data to make a “presumptive” model regarding contacts between rock units using the levels of the uncertainty rating scale.

Fourteen students either used only model or data uncertainty in their final report. Six students reference only data uncertainty ratings, eight students reference only model uncertainty ratings. Students who reported data uncertainty tended to mention concerns of accuracy and limited amount of data. For example, one student wrote:

“Further analysis of the faulting in the area revealed that some of the faults mapped do not have enough data at the level of evidence as presumptive or compelling. Due to these contradictions, some faults were left out, and a few faults with higher levels of evidence were included.”

This student did not assign uncertainty ratings to their models, but they did connect their models to the uncertainty ratings provided for the data during the course. They also showcase the idea that more data is needed to attain a classification at higher levels of the uncertainty scale. For students who reported only model uncertainty, this lack of data was also a concern; however, the quality of the data was not included in their explanations. We see this student use the uncertainty rating scale to support their need for more data to bolster claims they can make.

For many students who did not use the uncertainty rating scales in their final report, uncertainty was seen as problematic, rather than an inherent part of doing science. In the following example, a student reports that the older maps were “flawed”, and their new map is “unbiased.”

“The geologic maps that were provided were *flawed*. This leads to uncertainty in the mapping data. This means that data that was provided has uncertainty which then is incorporated in the new constructed map. [...] This project created an unbiased map of the Sage Hen Flat which can be used now for further research in the Sage Hen Flat area.”

For this student, the incorporation of newer data with technology and tools rendered it more trustworthy than the previous maps based on dated data collection techniques. Instead of seeing the evolving face of science in the maps, they saw a binary quality—biased and unbiased maps. Creating a binary within the interpretation as biased or unbiased meant that the student geologists had not yet grasped being a geologist, and how their experiences, knowledge, and skills influence the way they practice science (Oreskes, 2015). The uncertainty rating scale did not appear to influence these students’ thinking about the role of uncertainty in geoscience as a natural and necessary part of the practice of science, instead they maintained a view of science that was contrary to the goals of the uncertainty module, such that science was right or wrong, rather than fluid.

We revisited the reports of the students who did not reference uncertainty (10/32) to try to understand the lack of transfer of uncertainty in the module to uncertainty in their project report. The majority of students (9/10) made explicit reference to the accuracy or credibility of the data. For example, the following student never discusses uncertainty explicitly in their final report, but recognizes the tentative nature of science, a reason to teach scientific uncertainty to students.

“In conclusion, the gravity and AMS data along with field observations [...] have been useful in constraining the pluton subsurface geometry and deciphering the motion of faulting. This does not discredit either published maps, but rather serve as an example of how models or hypotheses change as new data is collected.”

Only one student did not explicitly mention either accuracy or credibility, stating,

“However, the gravity data does not register below 100 meters of thickness and does include places where the granite has been covered over with country rock due to faulting. Therefore the proposed contact lines are still plausible when they differ from the gravity data.”

This student uses hedging language (plausible) to temper the fixed nature of their claims which has been linked to uncertainty in the literature (Jordan et al., 2012). This term is used in the uncertainty rating scales, but the student makes no mention of uncertainty or the rating scales in their explanation, so we labeled this final report as “implicit uncertainty—hedging language”.

When collating the two sets of data, there were no clear patterns as to who requested uncertainty information in the end of course assessment of uncertainty and who used uncertainty ratings in their final report. Nine students requested to know about the uncertainty of geologist’s data or model in responses to the end of course assessment of uncertainty. Six of those students did not mention the uncertainty rating scale in their final report, and only one of those students explicitly references uncertainty in their final report. However, we do see an increase in requests to see the raw data and recognize that students who asked for raw data, but not uncertainty ratings, may have been looking to ascertain uncertainty by examining the source in the absence of uncertainty ratings.

## Discussion

Introducing uncertainty in science education offers an opportunity for fostering authentic scientific practices such as development of scientific explanations and argumentation. This study explores the ways explicit instruction in data and model uncertainty informed undergraduate geoscientists’ explanation of phenomena, in this context, a complex geologic phenomenon in an online geology capstone course. Our findings showcase that uncertainty can be explicitly taught in ways that promote students’ engagement in the practices of science that is inclusive of uncertainty. Support is evident from three points. First, the data from students’ assessments provided a window into their thinking about uncertainty, and specifically, a shift in thinking from uncertainty as lying within the data to being present throughout the practice of science. Secondly, providing students with examples of professional scientists wrestling with uncertainty in videos, their scientific process made public (Miller et al., 2018; Stroupe, 2014), exposed students to the social nature of science (Oreskes, 2015). The novice geologists could now see that the professional geologists did not discuss uncertainty as “bad” or fixable, but just part of the process. For many students, this likely transferred to their arguments in placing elements on their geologic map. Finally, students bolstered their arguments with consideration of their ability to assess uncertainty of data and models, a large part of doing science (Phillips et al., 2018; Sinatra et al., 2014) and the development of scientific literacy (Chen & Klahr, 1999; Kirch, 2012; Manz & Suárez, 2018; Metz, 2004). From these findings, two discussion points emerge that are important for science education: the role of uncertainty in the practice of geoscience, and the role of uncertainty in K-12 science education.

### Uncertainty in the practice of geoscience

Students’ experience with the uncertainty module provided them a way to think about uncertainty as an inherent part of the practice of science. Students benefit from approaching science as something they are doing, practicing science, rather than something they are learning about (Jiménez-Aleixandre et al., 2000), which was an integral part of the uncertainty module. In the module, we designed the components to engage students in first- and second-hand experiences of uncertainty within the practice of science. As a result, in post module assessments of uncertainty, students increasingly asked for information about geologic uncertainty and the data collected in order to assess differences in claims made by expert geologists. They were less interested in “fixing” the inherent uncertainty, and more interested in understanding it, accepting the uncertainty as part of the practice of geoscience. As geoscience is an hermeneutic science (Frodeman, 1995), knowing what went into a geoscientist’s interpretation can improve others’ understanding of the decision making process. In our module, we designed for this. The uncertainty rating scale provided students with a tool to describe where uncertainty existed and its extent. Learning modules provided video and narratives of professional scientists discussing uncertainty in data and models. The professional maps further showcased how even professionals can produce different models based on myriad factors. With these design features, student assessments post-course increasingly attributed differences in claims between geologists as related to uncertainty.

Students also showed understanding of this in crafting their explanations. In their final reports, students included uncertainty as part of the complex system, inherent in the process of doing science. One student positioned their understanding of the multiple models as changes in available evidence.

“I do not think both published maps are wrong, but rather the outcrop could have been exposed in more recent years due to weathering and erosion which could aid in visualizing the subsurface.”

Additionally, students frequently stated their own uncertainty in their claims, given the data available, “It is possible that these faults may also have normal components; there was no data that ruled out that possibility.” In their final reports, students were discussing uncertainty, both explicitly and implicitly, without a prompt specifically asking them to do so.

The uncertainty module also provided a way of making the scientific practices public, able to be scrutinized, and created a set of norms of practice (Kelly & Chen, 1999) through the use of the uncertainty rating scales. The uncertainty rating scale provided common terms to communicate uncertainty that was used by 16 of the 32 students in their final report. Grappling with uncertainty in complex phenomena is an integral part of engaging in scientific argumentation (Manz & Suárez, 2018) and making sense of science (Buck et al., 2014; Furtak & Penuel, 2019), such as students needed to do in their final report. We see room in future studies to examine the classroom discourse to further examine how these tools supported the sensemaking process that led to the final reports, the scientific arguments, produced by students.

### The role of uncertainty in K-12 science education

Although we focus here on burgeoning professional geologists, an understanding of the role of uncertainty in the practice of science does not need to start, nor should it start, at the undergraduate level. We must consider the role of K-12 science education in shaping a public understanding of the role of uncertainty. Though uncertainty is written into the Framework for K-12 Science Education (2012) and the Next Generation Science Standards (NGSS Lead States, 2013), the uptake by classroom teachers has not been immediate (Chen et al., 2019; Manz, 2015). This situation may result from uncertainty not explicitly being in the standards themselves, but rather implicit in the way some standards are written. Being direct and descriptive, as we see with the geoscience majors, helps students to acknowledge the uncertainty and incorporate it into their arguments. Furthermore, not only were these geoscience students crafting arguments, but they also critiqued the non-verbal arguments of maps crafted by other geoscientists. By introducing uncertainty, these critiques were modulated by the level of uncertainty in different aspects of the data, and thus more likely to avoid rejecting findings simply because there was some level of uncertainty. If we can support K-12 students to think critically about scientific arguments with consideration to the inherent place of uncertainty in the practice of science, we are more likely to develop a society in which decision making around science is grounded in evidence and reasoning.

## Limitations and Future Directions

This study presents three limitations. First, we focused solely on a geology context. We recognize that each science discipline will have its own unique challenges to documenting uncertainty in practice. However, we do feel that the rating scale will have cross-disciplinary implications. Therefore, a suggestion for future study would be to introduce this rating scale into new learning modules geared towards other geoscience and non-geoscience disciplines to see if an understanding of data and model uncertainty can be supported and be incorporated into novice scientists’ practice.

Second, this learning module has only been deployed in an online field course. Though our design intentions were for in-person, field-based instruction, the structure afforded ready conversion to the online module, which could be shared and used beyond our initial design. It is possible that sensemaking in virtual and in-person instruction may differ so radically that the module would have to be significantly revised for in-person learning environments. Contrasting learning about uncertainty in the two learning environments could help identify some of the constraints to online learning for practice-based courses. We plan to continue to collect information from the use of the module in new field courses (virtual and in-person) to reduce the uncertainty in our interpretations.

Third, it is conceivable that students would have developed an awareness of uncertainty and incorporated it in their write-up when presented with traditional materials without explicit discussion of uncertainty. While anecdotally this does not occur, a controlled contrast of business-as-usual instruction to the new uncertainty instruction materials would be required to confirm the magnitude of educational effects. We note that the authors would be ethically unwilling to conduct such a study given the awareness of uncertainty so clearly evident in students who were involved in this study.

Future research can also examine the ways in which articulation of uncertainty in explicit, reliable formats like the uncertainty rating scale impacts public understanding of uncertainty. By developing tools for communicating uncertainty between scientists that is successfully assimilated by students, we also support communication of uncertainty with the general public. Science cannot and does not exist in isolation. Rather, practitioners need to communicate with diverse communities about science in ways that would help the general public understand the uncertainty of science (Jeong et al., 2020). We must share our knowledge, and its limitations, with the world for the greater common good. To take full advantage of scientific research necessitates understanding the uncertainty in the science (Fischhoff & Davis, 2014). People who place too much confidence in uncertain science can face unexpected problems—failing to realize how wary they should have been, or the potential role of bias. In contrast, people who place too little confidence in science can miss opportunities and waste time and resources collecting data with no new value, or ignoring data that would have usefully guided future action. Creating space for all sciences to make clear their uncertainty and communicate it in comprehensible ways can improve decision making for all, not just scientists.

## Data Availability

Not applicable.
